# Non-growing/growing season non-uniform-warming increases precipitation use efficiency but reduces its temporal stability in an alpine meadow

**DOI:** 10.3389/fpls.2023.1090204

**Published:** 2023-01-27

**Authors:** Fusong Han, Chengqun Yu, Gang Fu

**Affiliations:** Lhasa Plateau Ecosystem Research Station, Key Laboratory of Ecosystem Network Observation and Modeling, Institute of Geographic Sciences and Natural Resources Research, Chinese Academy of Sciences, Beijing, China

**Keywords:** temperature sensitivity, temporal stability, trade-off, asymmetrical warming, Tibetan Plateau, alpine grassland

## Abstract

There are still uncertainties on the impacts of season-non-uniform-warming on plant precipitation use efficiency (PUE) and its temporal stability (PUE_stability_) in alpine areas. Here, we examined the changes of PUE and PUE_stability_ under two scenes of non-growing/growing season non-uniform-warming (i.e., GLNG: growing-season-warming lower than non-growing-season-warming; GHNG: growing-season-warming higher than non-growing-season-warming) based on a five-year non-uniform-warming of non-growing/growing season experiment. The GLNG treatment increased PUE by 38.70% and reduced PUE_stability_ by 50.47%, but the GHNG treatment did not change PUE and PUE_stability_. This finding was mainly due to the fact that the GLNG treatment had stronger influences on aboveground biomass (AGB), non-growing-season soil moisture (SM_NG_), temporal stability of AGB (AGB_stability_), temporal stability of non-growing-season air temperature (*T*_a_NG_stability_), temporal stability of growing-season vapor pressure deficit (VPD_G_stability_) and temporal stability of start of growing-season (SGS_stability_). Therefore, the warming scene with a higher non-growing-season-warming can have greater influences on PUE and PUE_stability_ than the warming scene with a higher growing-season-warming, and there were possibly trade-offs between plant PUE and PUE_stability_ under season-non-uniform-warming scenes in the alpine meadow.

## 1 Introduction

Precipitation use efficiency (PUE) of individual plant or plant community is not only one key component of resource use efficiency, but also an important indicator for carbon-water coupling mechanism of terrestrial ecosystems (Hu et al., 2010; Zhang et al., 2020). A large number of studies have examined vegetation PUE from various perspectives (Peterson et al., 1996; Paruelo et al., 1999; Lauenroth et al., 2000; Vermeire et al., 2009; Irmak, 2015; Busso et al., 2016), whereas there are still two uncertainties. Firstly, earlier papers related to PUE mainly focus on PUE itself and related driving factors rather than its temporal stability (PUE_stability_) (Hu et al., 2010; Li and Wu, 2016; Zhang et al., 2020; Sun et al., 2022). Plant PUE reflects the average capacity of plants to use precipitation. In contrast, PUE_stability_ mainly reflects the fluctuation and uncertainty of the capacity of plants to use precipitation (Sun et al., 2022). Plant PUE and PUE_stability_ can possibly have different correlations with environmental variables and different responses to external disturbance (e.g., climate warming) (Zhang et al., 2020; Sun et al., 2022). Compared with the responses of plant PUE to external disturbances, the responses of PUE_stability_ to external disturbances possibly have better guiding significance for ecosystem suitability management. Thus, it is not far from sufficient to study vegetation PUE only from plant PUE itself. It is necessary to strengthen the research on the PUE_stability_. Secondly, temperature is one of the key environmental factors affecting vegetation productivity and PUE (Zhang et al., 2020; Sun et al., 2022). The correlation between temperature and vegetation PUE is possibly an indicator of carbon-temperature-water coupling in terrestrial ecological systems. Elevated surface temperature is an indisputable issue (Fu and Sun, 2022). *In situ* warming experiment is one of the important techniques and even the best approach to study the influence of temperature on key factors and processes of ecosystem (Rustad et al., 2001; Klein et al., 2004). Compared with other important characteristic parameters of plants, studies on the influence of experimental warming on vegetation PUE are lacking at various spatial and temporal scales, let alone the PUE_stability_ (Alatalo et al., 2014; Fu et al., 2015; Barton and Schmitz, 2018). Current studies actually have focused on the response of plant water use efficiency at the leaf scale to experimental warming (Fu et al., 2015), which is not the response of vegetation PUE to experimental warming. More relevant studies are extremely necessary in order to solve the problems mentioned above.

Season-non-uniform-warming is an important feature of climate change over the Qinghai-Tibet Plateau (Wang et al., 2010). Under such scenes, several studies have tried to capture the influences of season-non-uniform-warming on alpine ecosystems on the Qinghai-Tibet Plateau (Liu et al., 2012; Ji et al., 2017; Zong et al., 2018; Fu and Shen, 2022). However, how such season-non-uniform-warming affects plant PUE and PUE_stability_ is still unknown. The Qinghai-Tibet Plateau as a whole is now warmer and wetter, but the inter-annual climate fluctuations are also likely to increase (Fu et al., 2022; Wang et al., 2022). It is still unclear whether plant productivity can capture the increasing climate fluctuation in alpine grassland ecological systems on the Qinghai-Tibet Plateau (Fu et al., 2018; Yu et al., 2019b). It is also still unclear whether warming and wetting directly affects productivity change in alpine grassland systems on the Qinghai-Tibet Plateau. These two aspects of uncertainties in turn further affects the high-quality and sustainable development of animal husbandry, the income of farmers and herdsmen, and even social stability and ethnic unity. This fact (i.e., the uncertainties on the influence of season-non-uniform-warming on plant PUE and PUE_stability_) limits our prediction of climate change impacts on alpine grassland systems. This fact also increases the uncertainty on adaptive management of alpine grassland systems under season-non-uniform-warming scenes. It is necessary to strengthen the studies on the impacts of season-non-uniform-warming on PUE and PUE_stability_ in alpine grassland systems on the Qinghai-Tibetan Plateau.

In this study, two non-uniform-warming circumstances of non-growing/growing season (i.e., GHNG treatment: warming level of growing-season higher than warming level of non-growing-season; GLNG treatment: warming level of growing-season lower than warming level of non-growing-season) were used to explore the influence of non-uniform-warming on alpine meadow PUE and PUE_stability_ of the Northern Tibet in 2015–2019. According to some previous studies (Fu et al., 2019; Fu and Shen, 2022), we hypothesized that experimental warming increased PUE but reduced PUE_stability_ (*H1*), and the GLNG treatment had greater influences on PUE and PUE_stability_ than the GHNG treatment (*H2*).

## 2 Materials and methods

### 2.1 Survey *area*, experiment design, data observation/simulation and the calculation of PUE and PUE_stability_

Earlier studies fully described the survey area, experiment design, measures/simulations of soil temperature (*T*_s_), air temperature (*T*_a_), soil moisture (SM), vapor pressure deficit (VPD), normalized difference vegetation index (NDVI), aboveground biomass (AGB), end of growing-season (EGS), start of growing-season (SGS) and growing-season length (GSL) (Fu et al., 2019; Fu and Shen, 2022). The survey area was illustrated in **Figure S1** and located in an alpine meadow of the Northern Tibet (Fu and Sun, 2022; Zha et al., 2022). Mean annual precipitation (MAP) was about 476.36 ± 96.69 mm and mean annual temperature (MAT) was about 1.96 ± 0.80 °C in 1963–2019 (Fu and Shen, 2022). The experiment plot area was about 20 m × 10 m. We used open top chambers (OTC) to elevate *T*_s_ and *T*_a_ in 2015–2019. There were three experiment treatments (i.e., CK: control, GHNG and GLNG) with three replicates. There were two types of OTC used in this experimental plot area. Both the top and bottom were hexagonal, each side of the open hexagon was 0.60 m, and the angle between the slope and the ground was 60°for both the two types of OTC. The vertical heights of the two types of OTC were 40 cm and 80 cm, respectively. For each one of the three replicates of the GHNG treatment, the 80 cm height OTC were set up from June to September, and the 40 cm height OTC were set up from January to May and from October to December. For each one of the three replicates of the GLNG treatment, the 40 cm height OTC were set up from June to September, and the 80 cm height OTC were set up from January to May and from October to December. For the ‘CK’ treatment, there were no OTC around all the year. All the *T*_s_, SM, *T*_a_ and relative humidity dataset were obtained from microclimate observation in 2015–2019. The VPD was estimated from observed *T*_a_ and relative humidity in 2015–2019. Monthly NDVI were observed by Tetracam Agricultural Digital Camera during the period of June-September in 2015–2019. Monthly AGB was simulated from observed NDVI during the period of June-September in 2015–2019 (equation 1) (Fu et al., 2013). The three variables of vegetation phenology were estimated from observed daily *T*_a_ in 2015–2019 (Fu and Shen, 2022). Earlier studies reported the influences of the GHNG and GLNG treatments on *T*_s_, SM, *T*_a_, VPD, AGB, EGS, SGS and GSL in 2015–2019 (Fu et al., 2019; Fu and Shen, 2022). We calculated PUE based on growing-season mean AGB and total precipitation (GSP) for each year of 2015–2019 (equation 2) (Sun et al., 2022). PUE_stability_ was the ratio of mean PUE to standard deviation of PUE (i.e., the ratio of mean_PUE to sd_PUE) from 2015 to 2019 (equation 3).


(1) $$AGB=10.33\times exp^{3.28\times NDVI}$$

(2) $$PUE=\frac{AGB}{GSP}$$

(3) $$PUE_{stability}=\frac{mean\_PUE}{sd\_PUE}$$

### 2.2 Statistical analyses

Duncan multiple comparisons, univariate regression analysis and random forest model were used. We calculated temporal stability for growing-season *T*_s_ (*T*_s_G_stability_), SM (SM_G_stability_), *T*_a_ (*T*_a_G_stability_) and VPD (VPD_G_stability_), non-growing-season *T*_s_ (*T*_s_NG_stability_), SM (SM_NG_stability_), *T*_a_ (*T*_a_NG_stability_) and VPD (VPD_NG_stability_), SGS (SGS_stability_), EGS (EGS_stability_), GSL (GSL_stability_), AGB (AGB_stability_) and PUE (PUE_stability_) in 2015–2019. Duncan multiple comparisons were analyzed for *T*_s_G_stability_, SM_G_stability_, *T*_a_G_stability_, VPD_G_stability_, *T*_s_NG_stability_, SM_NG_stability_, *T*_a_NG_stability_, VPD_NG_stability_, SGS_stability_, EGS_stability_, GSL_stability_, AGB_stability_, PUE and PUE_stability_ among the three treatments. We used univariate regression analysis to establish the relationship of PUE with warming duration, GSP, *T*_s_G_, SM_G_, *T*_a_G_, VPD_G_, *T*_s_NG_, SM_NG_, *T*_a_NG_, VPD_NG_, SGS, EGS, GSL and AGB, respectively (Zong and Fu, 2021). We also used univariate regression analysis to establish the relationship of PUE_stability_ with *T*_s_G_stability_, SM_G_stability_, *T*_a_G_stability_, VPD_G_stability_, *T*_s_NG_stability_, SM_NG_stability_, *T*_a_NG_stability_, VPD_NG_stability_, SGS_stability_, EGS_stability_, GSL_stability_ and AGB_stability_, respectively. We used random forest model to establish relative contribution of warming duration, GSP, *T*_s_G_, SM_G_, *T*_a_G_, VPD_G_, *T*_s_NG_, SM_NG_, *T*_a_NG_, VPD_NG_, SGS, EGS, GSL and AGB to PUE. We also used the random forest model to establish the relative contribution of *T*_s_G_stability_, SM_G_stability_, *T*_a_G_stability_, VPD_G_stability_, *T*_s_NG_stability_, SM_NG_stability_, *T*_a_NG_stability_, VPD_NG_stability_, SGS_stability_, EGS_stability_, GSL_stability_ and AGB_stability_ to PUE_stability_. The main packages of R 4.1.2 were agricolae, randomForest, rfPermute and ggpubr.

## 3 Results

**Figure 1:**
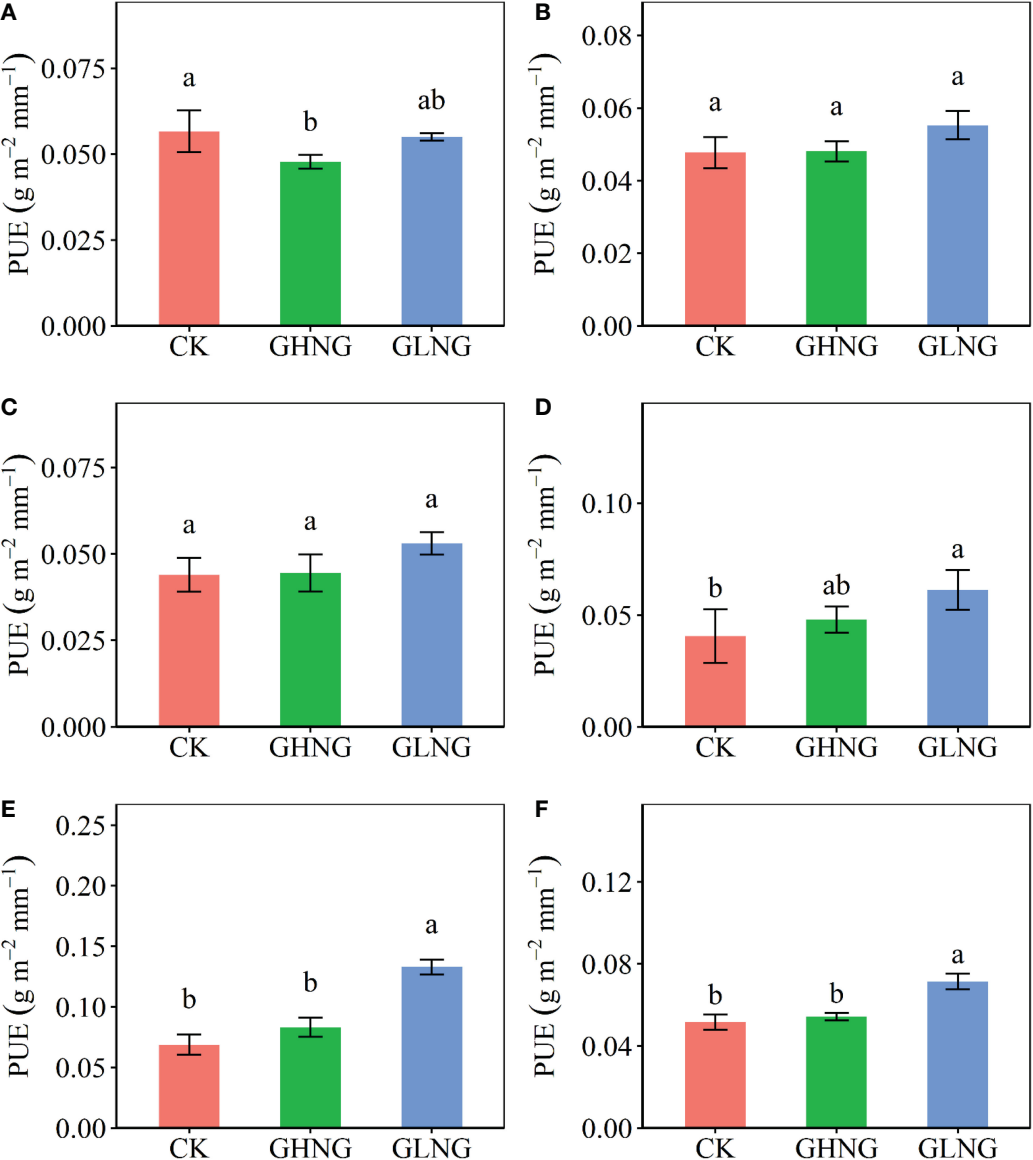
Comparison of precipitation use efficiency (PUE) among the control (CK), warming level of growing-season higher than warming level of non-growing-season treatment (GHNG) and warming level of growing-season lower than warming level of non-growing-season treatment (GLNG) in **(A)** 2015, **(B)** 2016, **(C)** 2017, **(D)** 2018, **(E)** 2019 and **(F)** 2015–2019, respectively. Different letters indicate significant differences among the three treatments at *p*<0.05 level.

**Figure 2:**
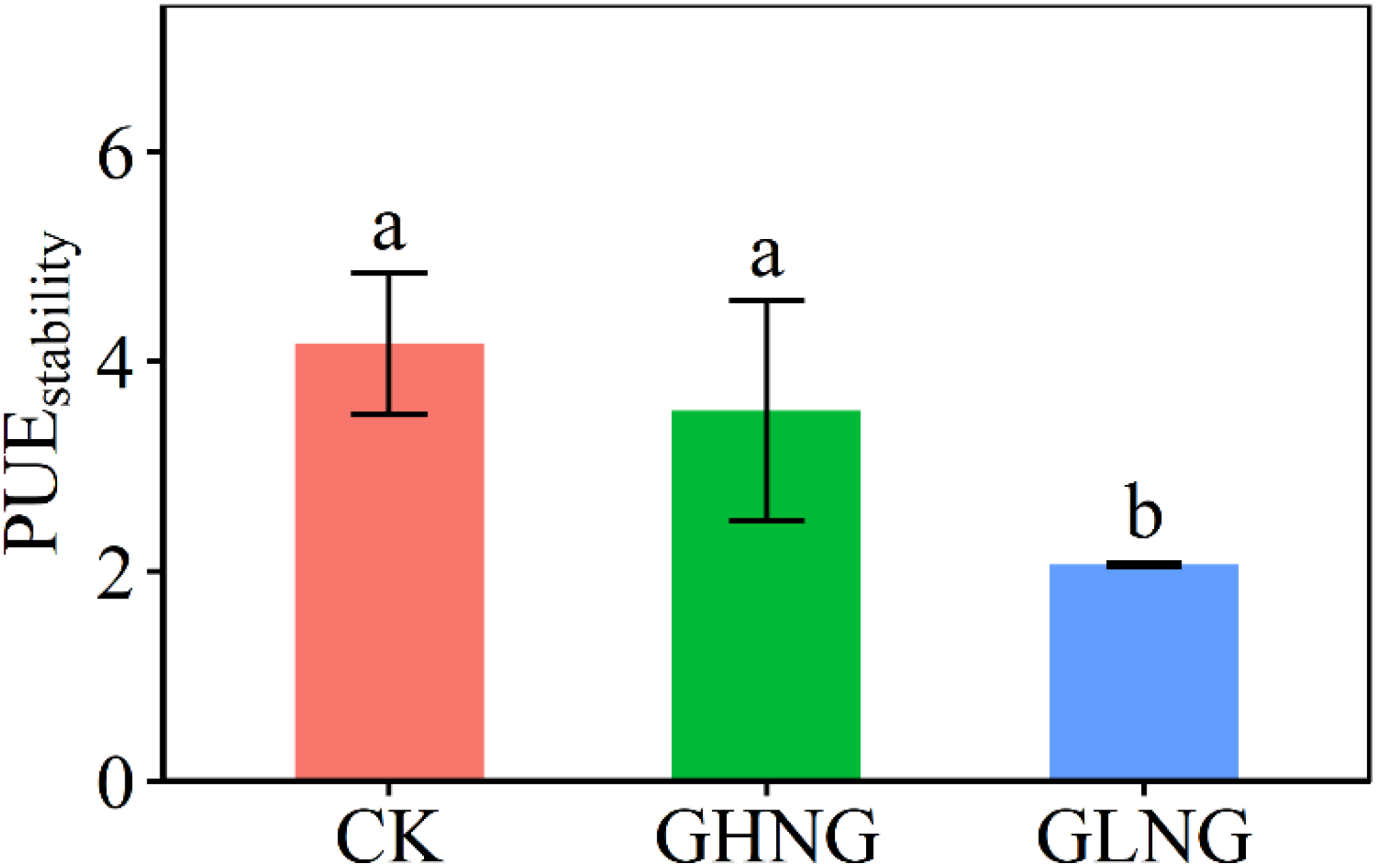
Comparison of temporal stability for precipitation use efficiency (PUE_stability_) in 2015–2019 among the control (CK), warming level of growing-season higher than warming level of non-growing-season treatment (GHNG) and warming level of growing-season lower than warming level of non-growing-season treatment (GLNG). Different letters indicate significant differences among the three treatments at *p*<0.05 level.

The GLNG treatment increased mean PUE by 38.70% across the five growing-seasons in 2015–2019 (**Figure 1**). The GLNG treatment increased PUE in 2018 by 50.71% and 2019 by 93.21%, but the GHNG treatment reduced PUE in 2015 by 15.62% (**Figure 1**). PUE of the GHNG treatment was 37.50% and 24.10% lower than that of the GLNG treatment in 2019 and 2015–2019, respectively (**Figure 1**). The GLNG treatment reduced PUE_stability_ by 50.47% (**Figure 2**).

**Figure 3:**
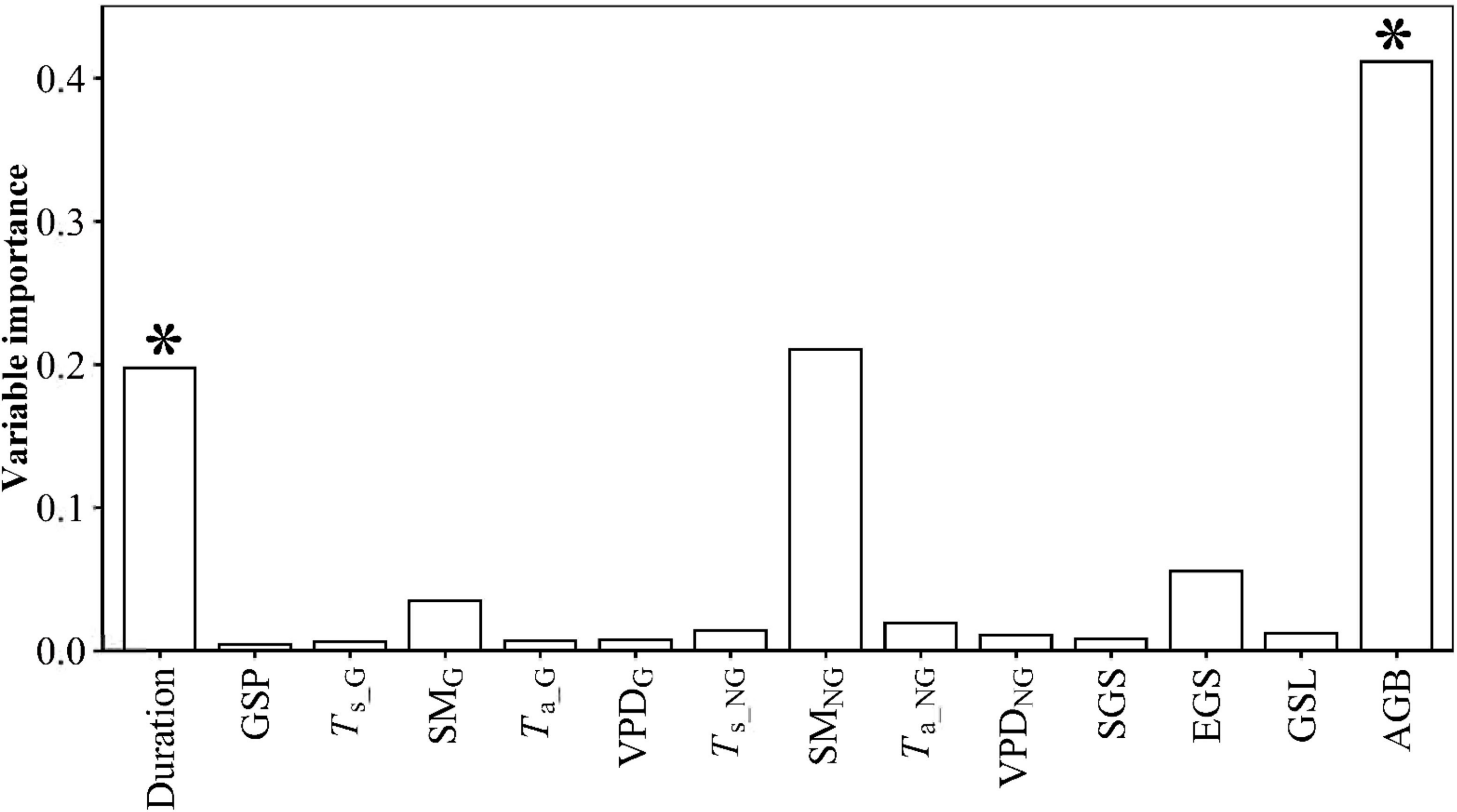
Relative contributions of warming duration (Duration), growing-season precipitation (GSP), growing-season mean soil temperature (*T*_s_G_), growing-season mean soil moisture (SM_G_), growing-season mean air temperature (*T*_a_G_), growing-season mean vapor pressure deficit (VPD_G_), non-growing-season mean soil temperature (*T*_s_NG_), non-growing-season mean soil moisture (SM_NG_), non-growing-season mean air temperature (*T*_a_NG_), non-growing-season mean vapor pressure deficit (VPD_NG_), start of growing-season (SGS), end of growing-season (EGS), growing-season length (GSL) and aboveground biomass (AGB) to precipitation use efficiency (PUE). * indicates this variable had a significant influence on PUE at *p*<0.05 level.

**Figure 4:**
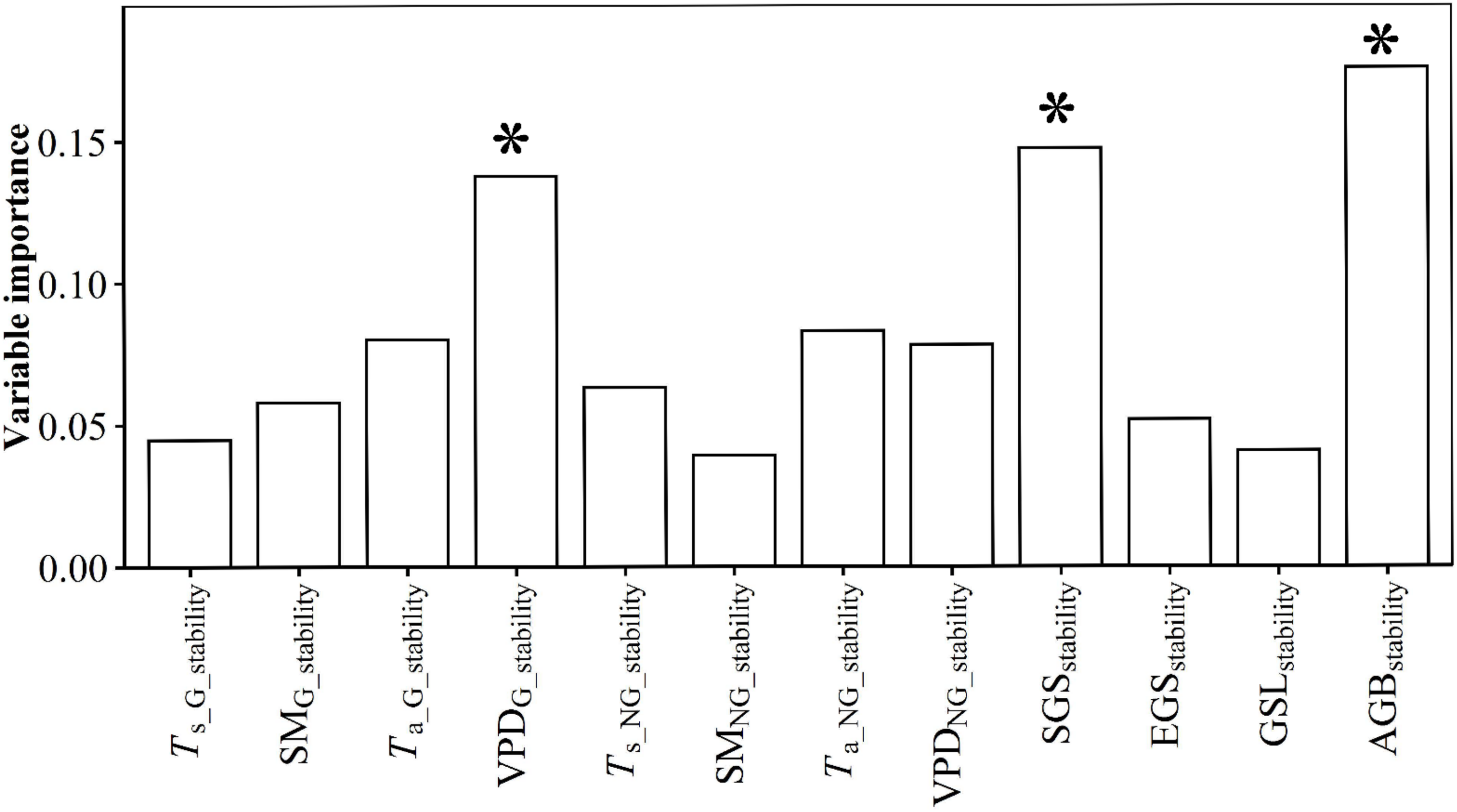
Relative contributions of the temporal stability of growing-season mean soil temperature (*T*_s_G_stability_), growing-season mean soil moisture (SM_G_stability_), growing-season mean air temperature (*T*_a_G_stability_), growing-season mean vapor pressure deficit (VPD_G_stability_), non-growing-season mean soil temperature (*T*_s_NG_stability_), non-growing-season mean soil moisture (SM_NG_stability_), non-growing-season mean air temperature (*T*_a_NG_stability_), non-growing-season mean vapor pressure deficit (VPD_NG_stability_), start of growing-season (SGS_stability_), end of growing-season (EGS_stability_), growing-season length (GSL_stability_) and aboveground biomass (AGB_stability_) to temporal stability of precipitation use efficiency (PUE_stability_). * indicates this variable had a significant influence on PUE_stability_ at *p*<0.05 level.

AGB and warming duration predominated PUE variation (**Figure 3**). PUE increased with increasing warming duration and AGB (**Figure S2**). AGB_stability_, SGS_stability_ and VPD_G_stability_ predominated the variation of PUE_stability_ (**Figure 4**). PUE_stability_ declined with VPD_G_stability_ and *T*_a_NG_stabliity_, but increased with SGS_stability_ (**Figure S3**).

The GLNG treatment increased *T*_a___stability_ by 129.33% and VPD_G___stability_ by 107.94%, but the GHNG treatment increased *T*_s___NG___stability_ by 267.15% (**Figure S4**). The GLNG treatment resulted in a greater increase in *T*_a___NG___stability_ than the GHNG treatment (**Figure S4**). The GLNG and GHNG treatments reduced SGS_stability_ by 47.30% and 36.01%, respectively (**Figure S5**). SGS_stability_ of the GHNG treatment was 21.42% greater than that of the GLNG treatment (**Figure S5**). The GHNG treatment increased EGS_stability_ by 237.31% (**Figure S5**). The GLNG treatment reduced AGB_stability_ by 56.24% (**Figure S5**).

## 4 Discussion

Our results denoted that water situations could have greater influences on PUE and PUE_stability_ than temperature situations. This event was in similar with some earlier researches which showed that water situations had stronger impacts on plant production (Fu et al., 2018; Zhang et al., 2021; Wang et al., 2022), soil respiration (Fu and Shen, 2022), PUE and PUE_stability_ (Sun et al., 2022) in alpine grasslands on the Tibetan Plateau, and water and carbon fluxes in grasslands external the Tibetan Plateau (Niu et al., 2008). Similar with earlier studies (Wang et al., 2022), water situations had a stronger influence on PUE than phenological change. Therefore, beside climate warming and its influences on grassland ecosystems, we should pay attention to precipitation change and its influences on grassland ecosystems, even if on the Tibetan Plateau.

Our results denoted that the GLNG treatment had obvious influences on PUE and PUE_stability_. This finding was not consistent with some earlier studies which demonstrated that non-growing/growing season-uniform-warming did not have obvious influences on PUE and PUE_stability_ in an alpine meadow near this study (Sun et al., 2022), and on PUE in a mixed-grass prairie (Xu et al., 2013). The different findings denoted that non-growing/growing season uniform-warming and non-uniform-warming may have different influences on grassland ecosystems and alpine ecosystems (Natali et al., 2012; Grant et al., 2017), and might be due to at least one of the succeeding causes. Firstly, the response of grassland ecosystems to warming might be linked with warming duration (Fu and Shen, 2022). Warming duration in this study and in Xu et al (Xu et al., 2013). was<5 years, but that of Sun et al (Sun et al., 2022). was >5 years (Sun et al., 2022). Secondly, warming magnitude might affect the response of grassland ecosystems to warming (Fu et al., 2018; Quan et al., 2020; Fu and Sun, 2022; Fu et al., 2022). Warming magnitude of Sun et al (Sun et al., 2022). was the lowest, and that of Xu et al (Xu et al., 2013). was the highest among the three studies. Thirdly, season-uniform-warming may underestimate season-non-uniform-warming influences on plant production (Zong et al., 2018; Wang et al., 2021; Fu and Shen, 2022) and in turn PUE. AGB was increased under the GLNG treatment, but non-significant impacts of season-uniform-warming on AGB were observed by these two earlier studies (Xu et al., 2013; Fu and Shen, 2022; Sun et al., 2022). Fourthly, the warming impacts on ecosystem structures and functions can vary with grassland types (Yu et al., 2019a; Fu and Sun, 2022). The grassland type was an alpine meadow for this study and Sun et al (Sun et al., 2022), but a mixed-grass prairie for Xu et al (Xu et al., 2013).

Consistent with the first hypothesis (*H1*), the GLNG treatment increased PUE but reduced PUE_stability_. This phenomenon warned that warming will likely to increase PUE at the expense of PUE_stability_. This event meant that although warming possibly increased the average capacity of plant productivity (here AGB) to utilize precipitation, it possibly decreased the capacity of plant production to adequately capture interannual variability in precipitation under season-non-uniform-warming conditions (Fu and Shen, 2022). PUE_stability_ and temporal stability of plant productivity will affect temporal stability of grassland carrying capacity and animal husbandry development. Reduced PUE_stability_ and AGB may in turn cause the increase in the risk of animal husbandry development. Compared to long-term (1963–2019) temporal stability (i.e., 4.61) of growing-season precipitation, temporal stability (i.e., 3.61) of growing-season precipitation during the recent five years from 2015–2019 decreased in the study area. This fact may further increase the uncertainty of plant production and the risk of animal husbandry development, at least for the alpine grasslands around the study area.

Consistent with the second hypothesis (*H2*), the influence degrees of the GLNG treatment on PUE and PUE_stability_ were stronger than those of the GHNG treatment. This event may be because of at least one of the succeeding causes. Firstly, AGB and AGB_stability_ was one of the variables in predominating the variation of PUE and PUE_stability_, respectively (**Figures 3** **,** **4**). The influence degrees of the GLNG treatment on AGB and AGB_stability_ were stronger than those of the GHNG treatment (Fu et al., 2019; Fu and Shen, 2022). Secondly, SM_NG_ had a certain positive influence on PUE (**Figures 3**, **S2**). The GLNG treatment had a stronger negative influence on SM_NG_ than the GHNG treatment (Fu and Shen, 2022). This fact can dampen the positive effect of the GLNG treatment on PUE. Thirdly, VPD_G_stability_ and SGS_stability_ negatively and positively predominated PUE_stability_, respectively (**Figures 4**, **S3**). Compared to the GHNG treatment, the GLNG treatment had a stronger positive influence on VPD_G_stability_, but negative influence on SGS_stability_ (**Figures S4**, **S5**). Fourthly, *T*_a_NG_stability_ had a certain negative influence on PUE_stability_ (**Figures 4**, **S3**), and the GLNG treatment had a stronger positive influence on *T*_a_NG_stability_ than the GHNG treatment (**Figure S4**). Fifthly, the GHNG treatment had stronger positive influences on *T*_s_NG_stability_ and EGS_stability_ than the GLNG treatment (**Figures S4**, **S5**). However, PUE_stability_ was not correlated with *T*_s_NG_stability_ and EGS_stability_ (**Figure S3**).

## 5 Conclusion

In summary, there was a trade-off between plant PUE and PUE_stability_ under season-non-uniform-warming scenes. Non-uniform-warming scene with a greater non-growing-season-warming than non-uniform-warming scene with a greater growing-season-warming had greater influences on plant PUE and PUE_stability_. Alpine plants may capture the inter-annual variation of precipitation to promote their own biomass accumulation under the future season-non-uniform-warming scenes. These findings can provide some theoretical and practical guidance for development of livestock husbandry in alpine grassland systems, at least in Tibet.

## Data availability statement

The original contributions presented in the study are included in the article/**Supplementary Material**. Further inquiries can be directed to the corresponding author.

## Author contributions

Conceptualization, GF and FH; methodology, GF; software, CY; validation, GF; formal analysis, GF and FH; investigation, GF; resources, CY; data curation, GF; writing—original draft preparation, GF and FH; writing—review and editing, GF and FH; visualization, FH; supervision, FH; project administration, CY; funding acquisition, CY. All authors contributed to the article and approved the submitted version.
