# Supplementary material for: Non-growing/growing season non-uniform-warming increases precipitation use efficiency but reduces its temporal stability in an alpine meadow
Source: Front Plant Sci. 2023 Jan 27;14:1090204. doi: 10.3389/fpls.2023.1090204 (PMC9911657; doi:10.3389/fpls.2023.1090204)
Supplement: Supplementary file 1 [file DataSheet_1.docx]

**Supplementary materials**


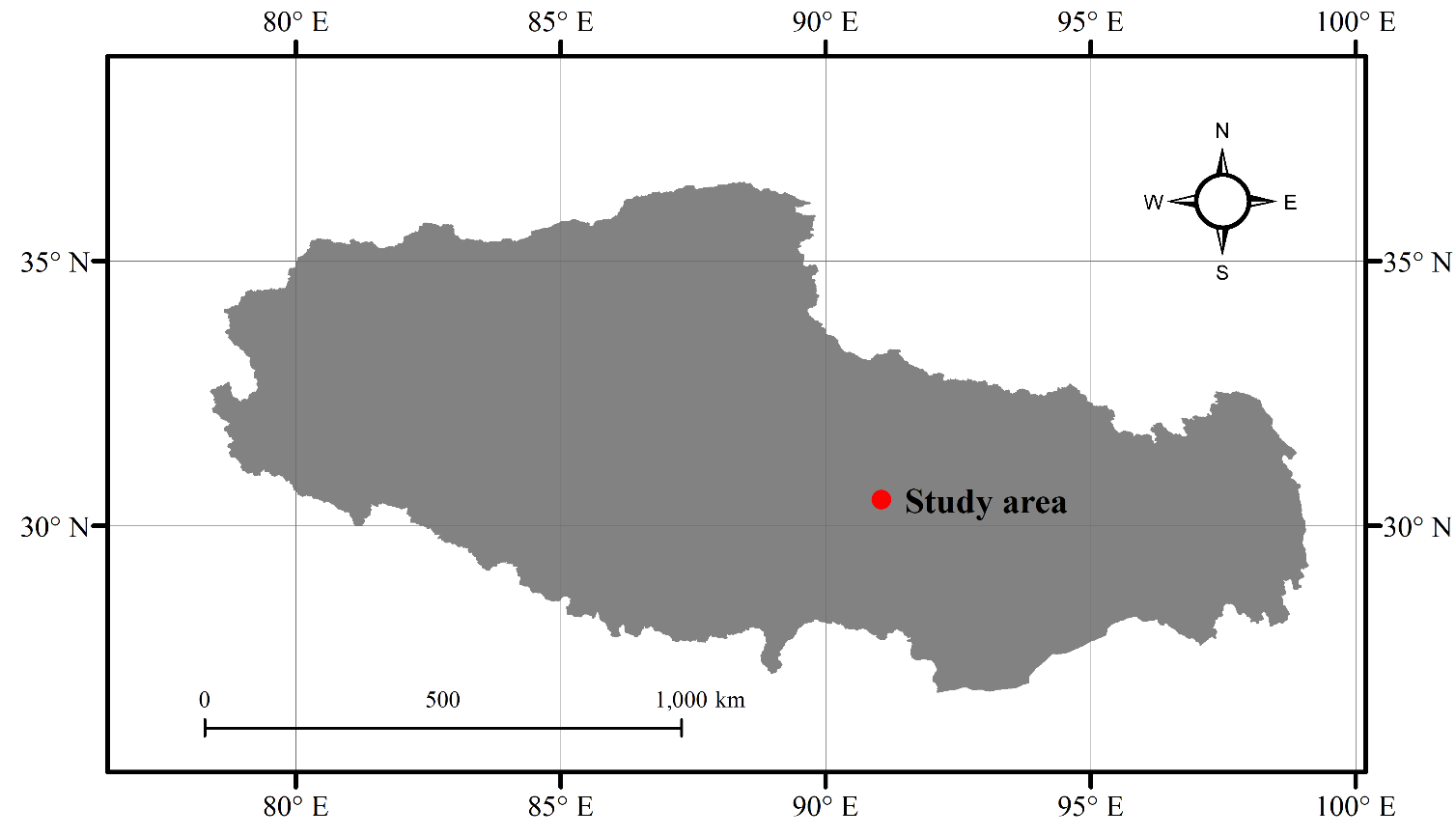


**Figure S1**. Geographical location of study area.


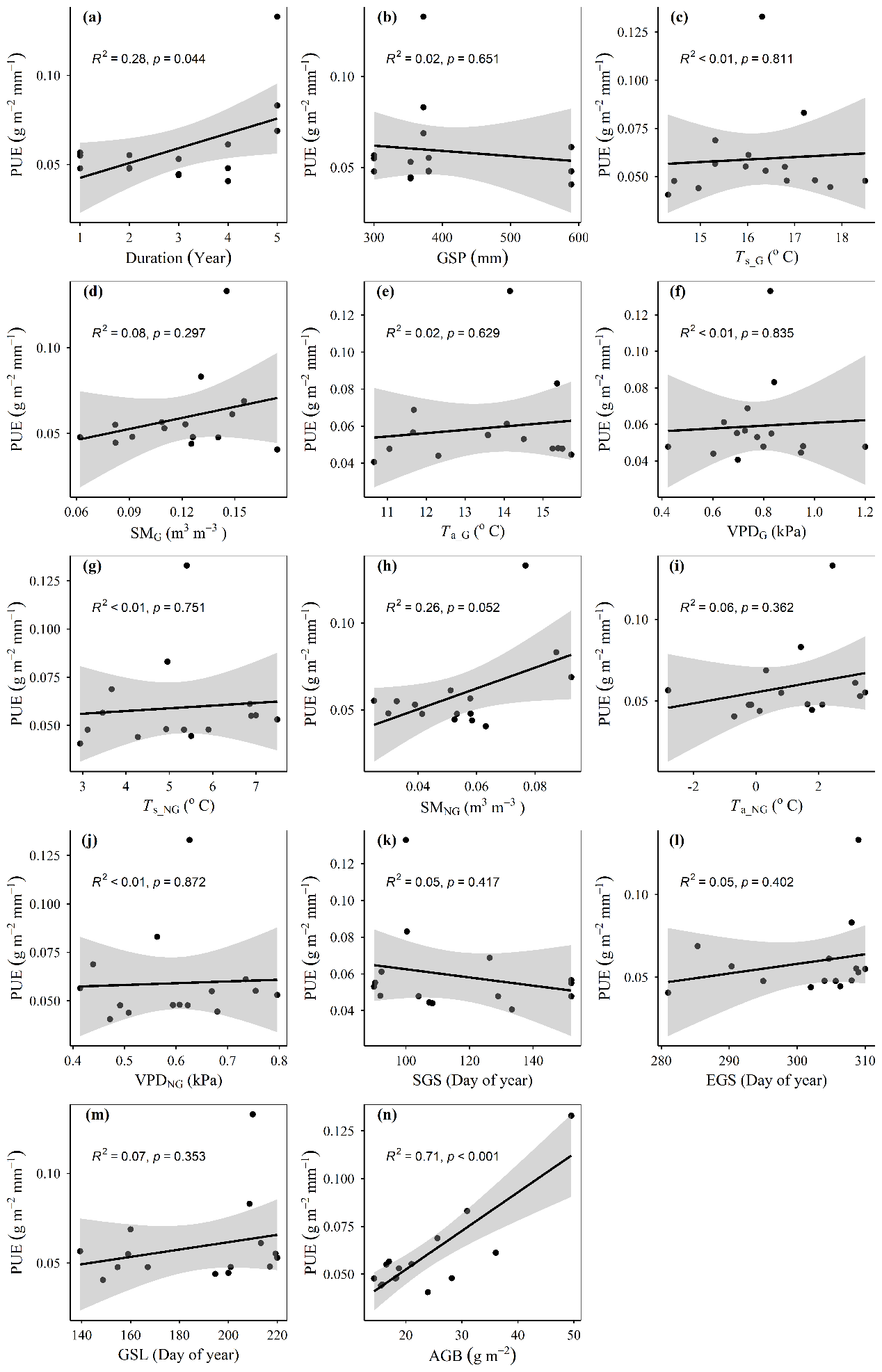


**Figure S2**. Relationships of precipitation use efficiency (PUE, g m^-2^ mm^-1^) with (a) warming duration, (b) growing-season precipitation (GSP), (c) growing-season soil temperature (*T*_s_G_), (d) growing-season soil moisture (SM_G_), (e) growing-season air temperature (*T*_a_G_), (f) growing-season vapor pressure deficit (VPD_G_), (g) non-growing-season soil temperature (*T*_s_NG_), (h) non-growing-season soil moisture (SM_NG_), (i) non-growing-season air temperature (*T*_a_NG_), (j) non-growing-season vapor pressure deficit (VPD_NG_), (k) start of growing-season (SGS), (l) end of growing-season (EGS), (m) growing-season length (GSL) and (n) aboveground biomass (AGB), respectively.


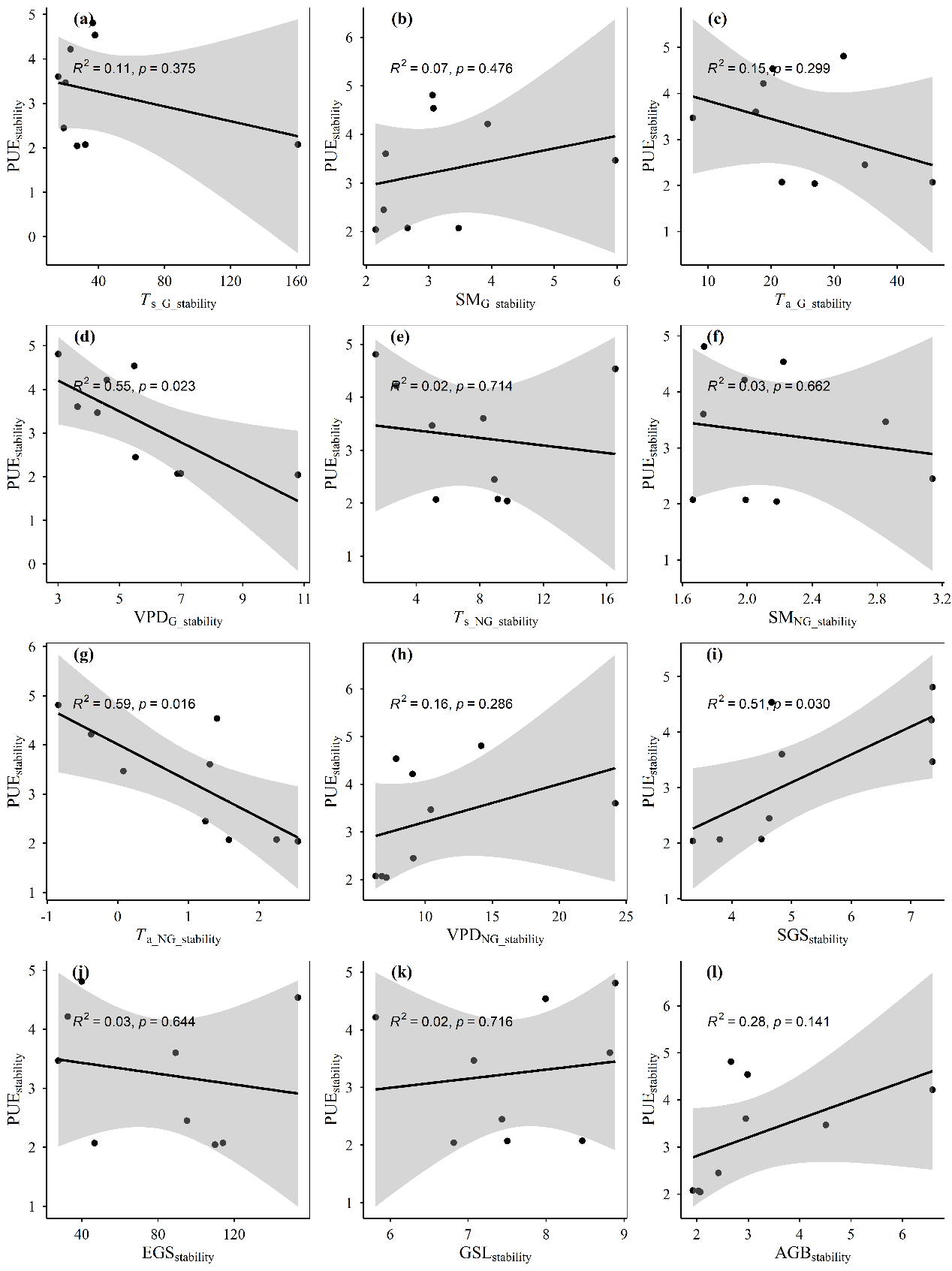


**Figure S3**. Relationships of temporal stability for precipitation use efficiency (PUE_stability_) with temporal stability for (a) growing-season soil temperature (*T*_s_G_stability_), (b) growing-season soil moisture (SM_G_stability_), (c) growing-season air temperature (*T*_a_G_stability_), (d) growing-season vapor pressure deficit (VPD_G_stability_), (e) non-growing-season soil temperature (*T*_s_NG_stability_), (f) non-growing-season soil moisture (SM_NG_stability_), (g) non-growing-season air temperature (*T*_a_NG_stability_), (h) non-growing-season vapor pressure deficit (VPD_NG_stability_), (i) start of growing-season (SGS_stability_), (j) end of growing-season (EGS_stability_), (k) growing-season length (GSL_stability_) and (l) aboveground biomass (AGB_stability_), respectively.


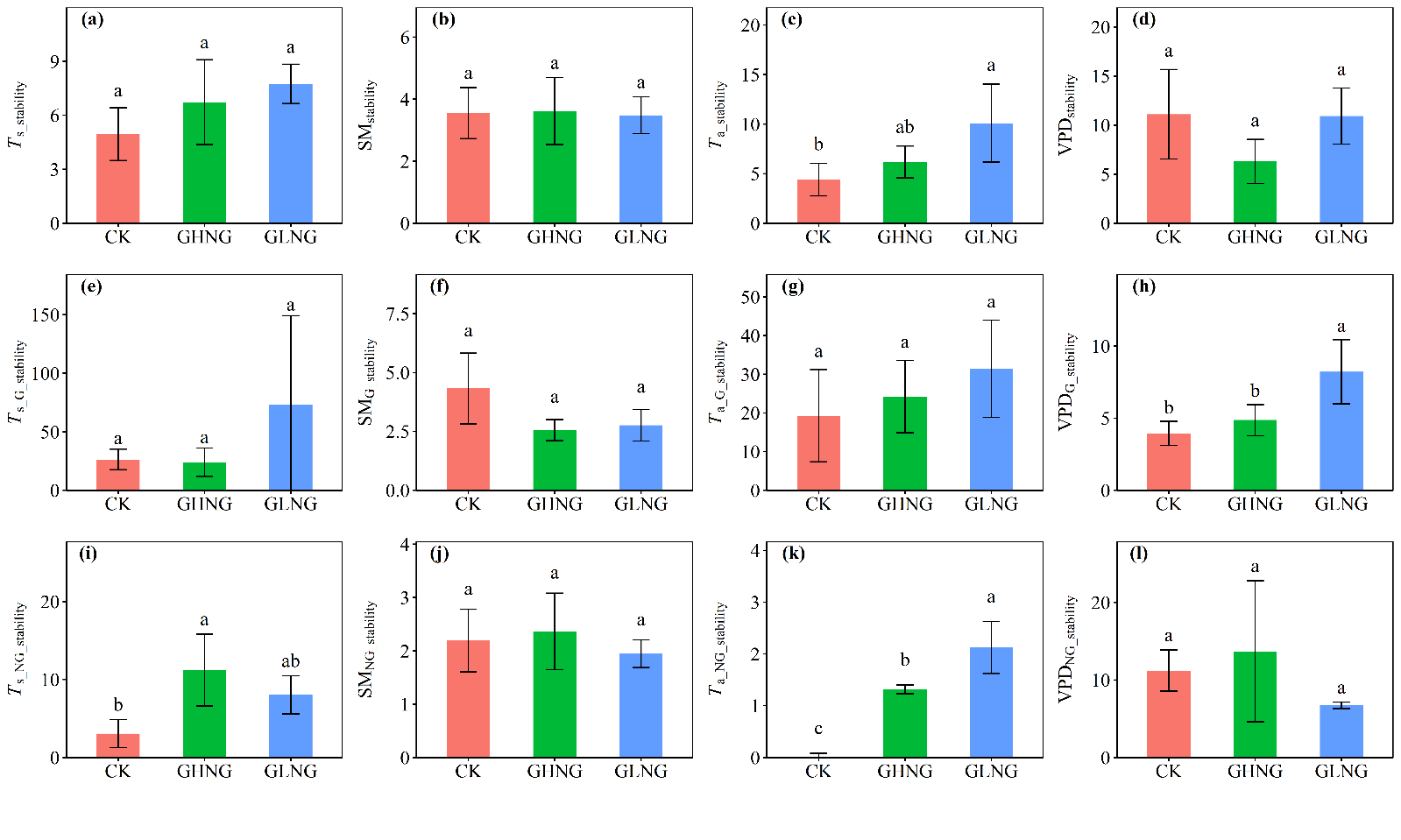


**Figure S4**. Comparison of temporal stability for (a) annual mean soil temperature (*T*_s___stability_), (b) annual mean soil moisture (SM_stability_), (c) annual mean air temperature (*T*_a___stability_), (d) annual mean vapor pressure deficit (VPD_stability_), (e) growing-season mean soil temperature (*T*_s_G_stability_), (f) growing-season mean soil moisture (SM_G_stability_), (g) growing-season mean air temperature (*T*_a_G_stability_), (h) growing-season mean vapor pressure deficit (VPD_G_stability_), (i) non-growing-season mean soil temperature (*T*_s_NG_stability_), (j) non-growing-season mean soil moisture (SM_NG_stability_), (k) non-growing-season mean air temperature (*T*_a_NG_stability_) and (l) non-growing-season mean vapor pressure deficit (VPD_NG_stability_) among the control (CK), warming level of growing-season higher than warming level of non-growing-season treatment (GHNG) and warming level of growing-season lower than warming level of non-growing-season treatment (GLNG), respectively. Different letters indicate significant differences among the three treatments at *p*<0.05 level.


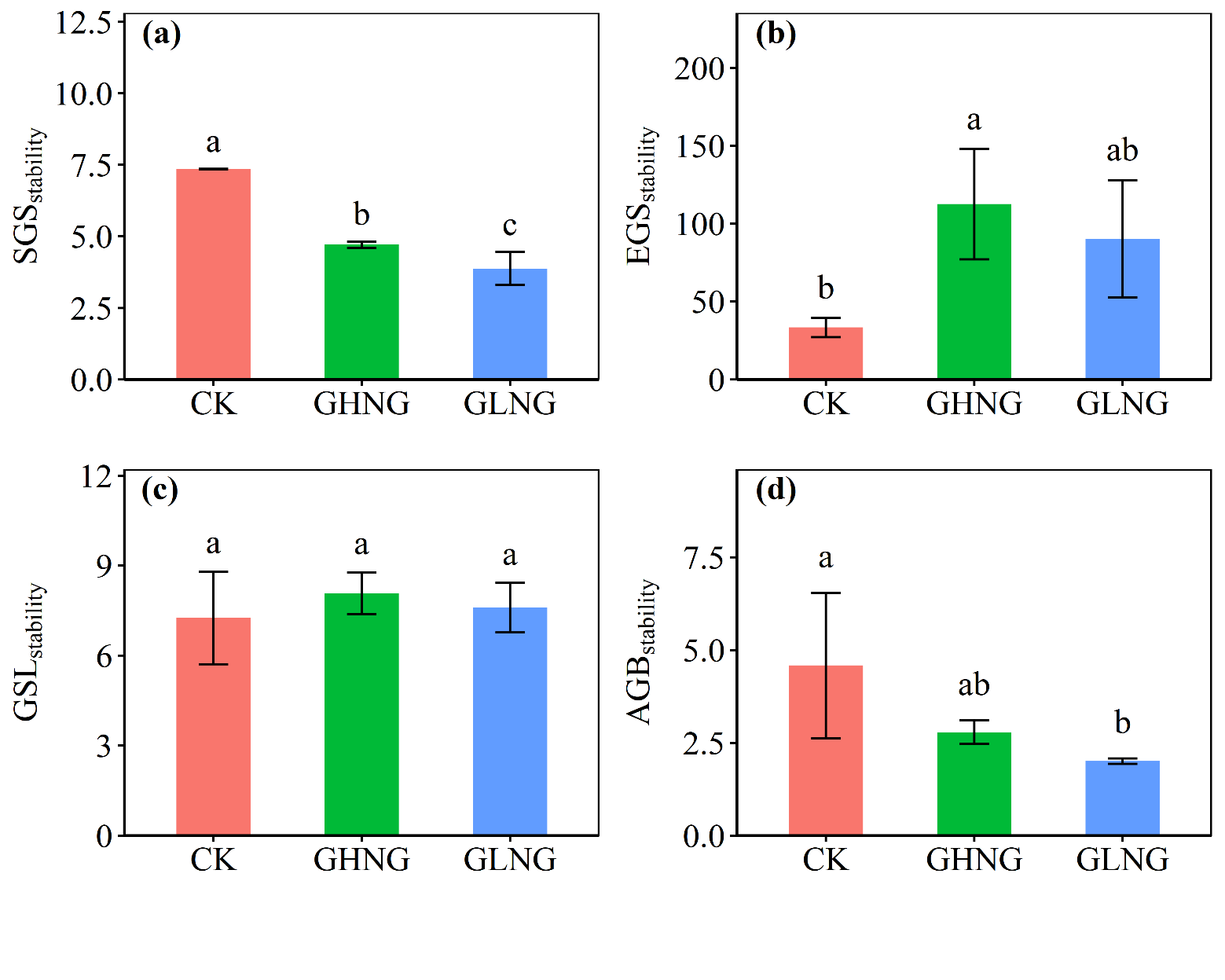


**Figure S5**. Comparison of temporal stability for (a) start of growing-season (SGS_stability_), (b) end of growing-season (EGS_stability_), (c) growing-season length (GSL_stability_) and (d) aboveground biomass (AGB_stability_) among the control (CK), warming level of growing-season higher than warming level of non-growing-season treatment (GHNG) and warming level of growing-season lower than warming level of non-growing-season treatment (GLNG), respectively. Different letters indicate significant differences among the three treatments at *p*<0.05 level.
